# Causes of male sexual trait divergence in introduced populations of guppies

**DOI:** 10.1111/jeb.12313

**Published:** 2014-01-23

**Authors:** A K Lindholm, M L Head, R C Brooks, L A Rollins, F C Ingleby, S R K Zajitschek

**Affiliations:** * Evolution & Ecology Research Centre, School of Biological, Earth and Environmental Sciences, University of New South WalesSydney, NSW, Australia; † Institute of Evolutionary Biology and Environmental Studies, University of ZurichZurich, Switzerland; ‡ Centre for Ecology, Conservation and Biosciences, College of Life and Environmental Sciences, University of ExeterPenryn, Cornwall, UK; § School of Life and Environmental Sciences, Deakin UniversityGeelong, VIC, Australia; ¶ School of Life Sciences, University of SussexFalmer, Brighton, UK; ** Department of Biology, The George Washington UniversityWashington, DC, USA

**Keywords:** alignment, coloration, genetic drift, introduced populations, natural selection, *Poecilia reticulata*, population divergence, predation, selection gradient, sexual selection

## Abstract

Males from different populations of the same species often differ in their sexually selected traits. Variation in sexually selected traits can be attributed to sexual selection if phenotypic divergence matches the direction of sexual selection gradients among populations. However, phenotypic divergence of sexually selected traits may also be influenced by other factors, such as natural selection and genetic constraints. Here, we document differences in male sexual traits among six introduced Australian populations of guppies and untangle the forces driving divergence in these sexually selected traits. Using an experimental approach, we found that male size, area of orange coloration, number of sperm per ejaculate and linear sexual selection gradients for male traits differed among populations. Within populations, a large mismatch between the direction of selection and male traits suggests that constraints may be important in preventing male traits from evolving in the direction of selection. Among populations, however, variation in sexual selection explained more than half of the differences in trait variation, suggesting that, despite within-population constraints, sexual selection has contributed to population divergence of male traits. Differences in sexual traits were also associated with predation risk and neutral genetic distance. Our study highlights the importance of sexual selection in trait divergence in introduced populations, despite the presence of constraining factors such as predation risk and evolutionary history.

## Introduction

Sexual selection is an important evolutionary process in natural populations and is often stronger than other forms of natural selection (Kingsolver *et al.*, 2001; Hoekstra *et al.*, 2002; Kingsolver & Pfennig, 2007; Svensson & Gosden, 2007). Variation among geographically isolated conspecific populations in sexual advertisement, mate choice and sexual behaviour is important because resulting differences in the direction and intensity of sexual selection may drive divergence in sexually selected and other correlated traits. Furthermore, covariation between male sexual advertisement and female preferences for those advertisements provides evidence that sexual selection can determine the direction and strength of evolutionary diversification (e.g. in orthopterans, *Ephippiger ephippiger*, Ritchie, 1991; house finches *Carpodacus mexicanus frontalis*, Hill, 1994; and frogs, *Physalaemus petersi*, Boul *et al.*, 2007). Thus, an examination of the patterns of interpopulation variation and covariation of sexual traits and sexual selection on those traits as well as the processes underlying divergence can help us to understand sexual selection, the evolution of mate choice and the potential for these processes to influence speciation (but see Claridge & Morgan, 1993; Houde, 1993; Verrell, 1999; Boughman, 2001).

Sexual selection is not the only process that influences the diversification of sexual traits. Other forms of selection can also impact sexually selected traits and preferences, which may lead to population-dependent trajectories of trait evolution that do not align with differences in sexual selection. For instance, antagonistic interactions between sexual and other forms of natural selection have been shown to influence the co-evolution of ornaments and preferences (Schwartz & Hendry, 2007; Gordon *et al.*, 2009; Weese *et al.*, 2010). Predation, in particular, exerts natural selection on both sexual advertisement traits and preferences for those traits. For example, sexually preferred males bearing exaggerated ornaments are also more conspicuous to predators (Endler, 1980; Magnhagen, 1991; Godin & McDonough, 2003; Millar *et al.*, 2006; Schwartz & Hendry, 2007). Net sexual selection can thus be weaker in the presence of predators (Schwartz & Hendry, 2007; Weese *et al.*, 2010). Indeed, predictable relationships between nonsexual and sexual selection like these can lead to parallel evolution of sexual traits and mate preferences as seen, for example, in guppies *Poecilia reticulata*, where males from low-predation sites developed larger body sizes and increasing coloration compared to males from high-predation sites, whereas females at high-predation sites discriminated against colourful males (Schwartz & Hendry, 2007). Similarly, in *Drosophila serrata*, changes in cuticular hydrocarbons (CHCs) induced by novel environments also led to divergence of female preferences for these CHCs among populations (Rundle *et al.*, 2005).

Genetic architecture can also influence the rate and direction at which sexual traits and preferences for these traits can evolve, leading to complex interactions between different forms of selection. For example, in an artificial selection experiment on male attractiveness in *Drosophila serrata* (Hine *et al.*, 2011), selection on a preferred trait led to high mating success, but only until an evolutionary limit had been reached. In other words, genetic constraints prevented the unlimited evolution of male sexual traits in the direction of sexual selection. Furthermore, the results highlighted the importance of the interplay between sexual and nonsexual fitness for the evolution of sexual traits and indicated that sexual selection alone (without additional factors such as changes in the environment or changing female preference) is unlikely to drive trait divergence.

The genetic variance–covariance matrix underlying sexual traits and mate preferences may influence the trajectory of trait divergence regardless of how selection operates (Harvey & Pagel, 1991; Schluter, 2000), and may lead to a mismatch between observed ornamental traits and sexually selected optima (Hine *et al.*, 2011). The strong influence of genetic constraints on the direction of divergence in sexually selected traits has been highlighted in a study examining nine *Drosophila serrata* populations (Chenoweth *et al.*, 2010). Chenoweth *et al*. found that sexual selection alone could only account for 10% in population divergence in male CHCs, due to the fact that genetic variation in male CHCs in the direction of sexual selection was low. The evolution of CHCs followed the axes of genetic variance rather than the direction of sexual selection.

Another important factor that may influence evolutionary trajectories is evolutionary history. However, as colonizing populations are often small, and subject to founder effects or bottlenecks, evolutionary change may also result from genetic drift and inbreeding, leading to the loss of genetic variation. Alternatively, interactions between genotype and the new environment, as well as the mixing of genetic variation, when individuals from multiple source populations are introduced to the new site can result in increased additive genetic variance and new patterns of multivariate genetic covariation (Kolbe *et al.*, 2004), which can have strong effects on the direction of evolutionary change post-introduction. Untangling these contributions to trait evolution should lead to a better understanding of both trait divergence and biological invasions.

Our aim in this study was to identify the factors that cause population divergence in male sexual traits. Specifically, we are interested in the roles of selection, drift and constraints. Replicated species introductions provide excellent opportunities to do this. Colonization of new habitats often leads to rapid trait divergence due to adaptation to novel selective environments (Arnold *et al.*, 2001; Reznick & Ghalambor, 2001; Hendry *et al.*, 2008). Previous studies of experimental introductions (Losos *et al.*, 1997; Reznick *et al.*, 1997) show that they can lead to predictable, rapid evolutionary diversification that may parallel diversification seen in native ranges. For example, transplantations of guppies, from different source populations to previously unoccupied neighbouring streams, led to the evolution of male traits along the trajectories allowed by differing predation regimes, taking the differences of the source populations in male traits into account (Endler, 1980). In contrast, other examples show diversification along different trajectories in introduced compared to source populations, such as in house sparrows *Passer domesticus*, which were introduced to North America from Europe and evolved latitudinal clines in body size which were opposite in direction to the clines in Europe (Johnston & Selander, 1973).

In our study, we examine male sexual trait evolution in populations of guppies introduced to Australia, a species known to show geographical covariation between male advertisement and female choice among naturally occurring populations. Among populations, there are complex multivariate differences in male ornamentation and in mating preferences (Endler, 1990, 1995; Houde & Hankes, 1997). The area of orange coloration and the number of black spots are each positively correlated with the strength of preferences that females express for these traits in natural populations (Houde & Endler, 1990; Endler & Houde, 1995). Conspicuous colour patterns are also associated with the incidence of visual predators (Endler, 1987; Schwartz & Hendry, 2007). Together, these studies provide empirical support for a match between the signalling environment, male display and female mate choice. However, the match between male display and female preference in guppies is neither perfect nor universal. For example, a comparison of female preferences between two populations differing strongly in male orange area found no differences in levels of female sexual responsiveness or orange preference functions (Houde & Hankes, 1997).

The fastest diversification of sexually selected traits ever observed in natural populations is that of male coloration in guppies (Endler, 1980; Svensson & Gosden, 2007). Life history traits in guppies can also evolve very rapidly (Reznick *et al.*, 1990) in response to altered selection when introduced to new streams within Trinidad and especially in response to modified predation regime (Millar *et al.*, 2006; Schwartz & Hendry, 2007; Gordon *et al.*, 2009). Feral guppy populations have become established in hundreds of natural water bodies around the world, due both to their proliferation as pets and to their perceived usefulness in mosquito control (Lindholm *et al.*, 2005). In North Queensland, Australia, several known introductions of guppies have occurred since 1910 (Lindholm *et al.*, 2005).

To document interpopulation variation in both male sexual traits and sexual selection on these traits, we collected males and females from six introduced populations and measured sexual selection in each of these populations in laboratory trials, using paternity analysis. We predicted that if sexual selection was important in determining interpopulation variation in male sexual traits, then observed divergence in these traits would covary with the direction of sexual selection gradients (Chenoweth *et al.*, 2010). We also tested whether predator-induced natural selection or genetic drift was associated with interpopulation variation in male display traits. If natural selection has been important in the divergence of male sexual traits, then we predicted that interpopulation variation would be associated with important ecological parameters such as predation intensity (measured here as the presence or absence of piscivorous fish). Alternatively if genetic drift is important in determining interpopulation variation in sexual traits, then we expected associations with either genetic (measured from population divergence at neutral genetic markers) or geographical distance.

## Materials and methods

**Figure 1:**
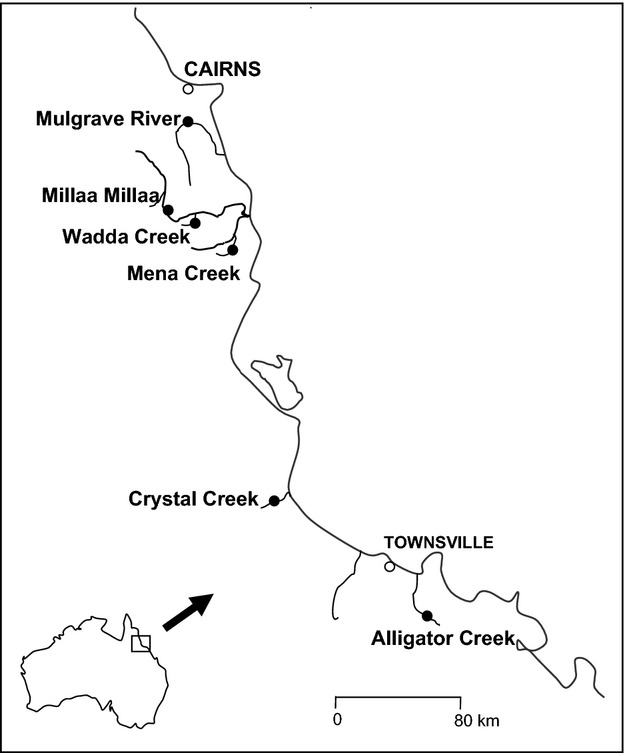
Sampling locations (black dots) of six feral guppy populations in northern Queensland, Australia

Adult male and female guppies were collected with permission from the Queensland Parks and Wildlife Services (Scientific Purposes permit F1/000428/01/SAA) using a dip-netting technique while wading in shallow water near the shore, consistently at all populations. Fish were collected from 5–12 April 2002 at the following sites in North Queensland, Australia: Alligator Creek (‘Ack’, 19.45°S, 146.97°E), Big Crystal Creek (‘Crc’, 18.98°S, 146.23°E), Mena Creek (‘Mnc’, 17.65°S, 145.97°E), the pond at the base of Millaa Millaa Falls (‘Mlm’, 17.50°S, 145.62°E), Mulgrave River (‘Ulg’, 17.12°S, 145.45°E) and Wadda Creek (‘Wdd’, 17.60°S, 145.83°E) (Fig.1). These populations stem from a minimum of two female source populations introduced at nonadjacent locations (Lindholm *et al.*, 2005). It is unknown when the populations originated, but guppies were first introduced into northern Queensland around 1910 (Lindholm *et al.*, 2005). The guppies were air-transported to Sydney, and populations were housed in separate large, widely spaced tanks in a greenhouse at the University of New South Wales. All fish were maintained on natural daylight schedules and fed live brine shrimp 5 days per week. All methods used in this experiment were approved by the UNSW Animal Care and Ethics Committee (clearance number 00/109).

### Mating trials and measuring male traits

Two weeks before mating trials commenced, males were removed in groups of ten. Each male was anaesthetized by immersion in a slurry of ice and photographed on its right side with a Nikon Coolpix 950 (Nikon, Tokyo, Japan) digital camera and fin-clipped at the distal end of the tail fin for the isolation of DNA. Males were then housed in individual tanks for 2 weeks, by which time tail fins had regrown. Sperm samples were taken on this day following the methods of Mathews *et al*. (Mathews *et al.*, 1997). Estimates of sperm number were square-root-transformed for parametric analysis.

Males were added in their groups of ten to 200-L aerated plastic tubs. Each tub was lined with gravel, decorated with plastic plants and two cinder blocks. On the day after males were added, ten females from the same population were weighed and measured and introduced to the tank, giving an equal sex ratio. We intended to set up three replicate mating tanks per population, but deaths in some populations limited us to two mating trials for Mulgrave River fish and to a third trial of only eight pairs from Millaa Millaa and five pairs from Mena Creek. After adding the females, we removed and discarded all offspring born for the next 5 weeks. As guppies are typically born after three or 4 weeks of gestation (Houde, 1997), any offspring born within this period are likely to have resulted from a brood cycle started before the mating trial. We then transferred females to individual tanks and waited for them to produce offspring. This allowed us to unambiguously match offspring to mother. Offspring were captured on their day of birth, killed and preserved in 70% ethanol until DNA extraction. After producing their first brood, or a minimum of three offspring, females were fin-clipped for DNA extraction.

Photographs of males were analysed using MeasureMaster Software (version 3.44 (+), 1999 Leading Edge Pty Ltd, Adelaide, Australia) and a digitization tablet. The areas of the body and tail were first measured, and then the areas of the body covered by black, fuzzy black, orange and total iridescence were measured, following the standard protocol (e.g. Head, 2005).

### Paternity analyses

DNA was isolated from all mothers, three of their offspring and all potential fathers, by salt precipitation, using Puregene Tissue Kit (Gentra, Gentra Systems, Minneapolis, MN, USA). Nine fluorescently labelled polymorphic microsatellite loci were amplified and scored using Genemapper software (Applied Biosystems, Foster City, CA, USA): TCTG and sat4 (Taylor, 1999), TTA (Taylor, 1999 with redesigned primers), KonD6, KonD15 and KonD21 (Seckinger *et al.*, 2002), Pr39 and Pr80 (Becher *et al.*, 2002) and Pr67 (Becher & Magurran, 2004). The average number of alleles per locus was 4.5 ± 0.25 (SE) per population.

Parent and offspring genotypes were analysed using Cervus 3.0 (Marshall *et al.*, 1998). As Cervus simulations assume Hardy–Weinberg equilibria and linkage equilibrium (Marshall *et al.*, 1998), we tested parental genotypes and found no deviation from Hardy–Weinberg expectations and no linkage disequilibrium, using Genepop web version 3.4 (http://wbiomed.curtin.edu.au/genepop/). Paternity analyses with one known parent were performed using the criteria of number of candidate sires equalling the number of males in a trial, proportion of candidate sires sampled equalling 1, allowing a 1% error rate, the observed proportion of loci typed (ranging from 0.99 to 1.0) and a 80% level of confidence.

### Population variation in male traits

To determine whether male phenotypes differed between the populations, we used multivariate analysis of variance (manova). Each male trait (four colour traits, sperm number, body and tail size, *N* = 7) was included as a response variable, and population was included as fixed factor.

### Population variation in sexual selection

For selection analyses, relative fitness was calculated as individual fitness (number of offspring sired by a given male) divided by mean fitness (average number of offspring per male) within each trial, and the seven male traits (outlined above) were standardized to the experiment-wide mean and standard deviation (Lande & Arnold, 1983) to allow comparison of the strength of selection both across different traits and across the different populations. To determine whether populations differed in linear sexual selection on male traits, we used a sequential model building approach (Draper & John, 1988). First, we fitted an ancova model containing population as a fixed effect and the linear effects of each of the male traits under investigation as covariates. This model was then compared to a model to which we added linear covariate by population interactions. We determined whether the addition of these interaction terms significantly improved the fit of the model using a partial *F*-test (Bowerman & O'Connell, 1990). When the addition of the interaction terms significantly improves the fit of the model, this indicates that linear sexual selection differs between the populations. We did not calculate nonlinear selection gradients because of sample size limitations.

### Within-population alignment of male trait variation and sexual selection

Knowing that both male traits and sexual selection differed between the populations, we wanted to establish whether sexual selection was driving population divergence in male traits and what other factors might be implicated in constraining the response of male traits to selection. To do this, we first calculated linear selection (*β*) vectors for each population. The linear selection vector for a given population is a vector of the seven linear selection gradients obtained from a multiple linear regression model (with relative fitness as the response variable and the seven mate traits as predictor variables). To look at the alignment of male trait variation with the direction of sexual selection within each population, we calculated the angle, *θ*, between the vector of directional selection, (*β*), and the vector of population mean values for the seven male traits for each pairwise comparison of populations using the following equation:
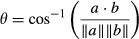
1

This was calculated separately for each population by substituting the vectors of interest into equation (1), such that *a* is the vector of population trait means and *b* is the vector of directional selection for a given population. To calculate 95% confidence intervals around each angle estimate, the relative fitness data were randomized within each population, and the selection gradients were re-estimated from the multiple regression models described above. This was repeated 1000 times to generate a distribution of 1000 angle estimates, from which a confidence interval was calculated. This angle, *θ*, gives a measure of how well-aligned selection and phenotypic variation are within each population. The directionality of the phenotypic vector in each of these calculations is not meaningful, and so we interpreted an angle of 90° as the maximum constraint, where the vector of selection is rotated orthogonal to the phenotypic vector, suggesting that there might be some form of constraint within that population preventing male traits from evolving in the direction of selection. For ease of interpretation, angles between 90° and 180° are represented as the equivalent angle between 0° and 90°.

### Among-population covariation in divergence of male traits and sexual selection

To compare population divergence in male traits with divergence in sexual selection, we followed the methods of Chenoweth *et al*. (2010). First, we created a **D** matrix which estimated the variance–covariance matrix among the six population means for each of the seven male traits. We then used the selection gradients (*β*) for each population obtained from the selection analysis described above to create a **B** matrix, which represents the variance–covariance matrix among the six population selection gradients (*β*) for each of the seven male traits.

To compare the orientation of these two matrices, we used the Krzanowski method (Krzanowski, 1979). This method required a principal component analysis of each matrix to determine the number of principal components needed to explain most of the variation in each matrix. Only principal components that had eigenvalues greater than one were used. This gave us two principal components for both matrices which in both cases explained over 90% of the variance. These two dimensional subspaces were then compared using equation (5) from Chenoweth *et al*. (2010). All analyses were conducted in R 2.15.0 (R Development Core Team, 2012).

### Identification of Predation regimes

We recorded fish species present at each of the collection sites at the time of collection (see also Head, 2005) and during snorkelling. These recordings revealed a total of eleven fish species present at our study sites, of which only three were considered to be potential predators of adult guppies (assessed blind to origin by J.A. Endler with the help of B. Pusey). Observed potential predators were the marbled eel (either *Anguilla obscura* or *Anguilla reinhardtii*), jungle perch (*Kuhlia rupestris*) and mangrove jack (*Lutjanus argentimaculatus*). *Anguilla* sp. and *K. rupestris* were recorded at both the Alligator and Crystal Creek sites, and *L. argentimaculatus* was also recorded at Crystal Creek. A large proportion of the diet of these species comprises small fish comparable in size to guppies (Pusey *et al.*, 2004). None of the predatory species were recorded at the remaining four sites. Due to our noninvasive sampling techniques, we cannot exclude the presence of predatory species at the sites that were classified as ‘no predation’; however, we believe that our sampling regime does provide a reliable estimate of relative predation intensity.

### The role of genetic and geographical distance in determining population variation in male traits and sexual selection

To determine whether genetic or geographical distance could account for any variation between populations in male traits, we employed a matrix comparison approach often used in population genetic studies (Geffen *et al.*, 2004; Ramachandran *et al.*, 2005). We also investigated whether the variation between populations in sexual selection itself was related to genetic or geographical distance. To do this, we calculated matrices of genetic and geographical distances within Genalex 6.2 (Peakall & Smouse 2006). We report the results based on Nei's genetic distance, but an analysis based on *F*_*ST*_ gives very similar results (not shown). Linear geographical distances were calculated based on latitude and longitude coordinates; these were highly correlated with estimated waterway distances (Spearman's rho = 0.94). We also calculated Euclidean distance matrices for male traits (using standardized trait population means) and sexual selection gradients (using population linear selection gradients for each trait obtained from the above selection analysis). The correlations between these matrices and their significance were calculated using Mantel tests (Sokal & Rohlf, 1995) in PopTools (Excel add-on). Significant correlations between male trait distance and genetic distance or geographical distance may act to constrain male response to sexual selection. On the other hand, significant correlations between sexual selection distance and genetic distance or geographical distance may indicate that genetic background limits the potential for sexual selection to drive trait divergence.

### The role of predation regime in determining population variation in male traits and sexual selection

Ecological differences between the populations may be important in determining the relationship between sexual selection and trait divergence. Predation regime has previously been shown to be important in shaping male traits that are also targeted by sexual selection (Endler, 1980; Houde, 1997; Ruell *et al.*, 2013). To test whether population differences in male traits or sexual selection on these traits were associated with population predation regime, we conducted analysis of variance, testing the effect of predation regime on each of the male traits measured (pooled to population means) as well as on each selection gradient associated with each of these traits. To control for the potential for increased type I error that is associated with conducting multiple tests, we calculated corrected *P*-values using the false discovery rate method proposed by Benjamini & Hochberg (1995).

## Results

### Paternity analyses

The proportion of males that were successful in siring offspring within a trial did not differ between the populations (binary GLM, *z* = 0.895, d.f. = 1.15, *P* = 0.37). The males siring offspring per trial ranged from 50% to 100%.

### Male traits vary among populations

**Figure 2:**
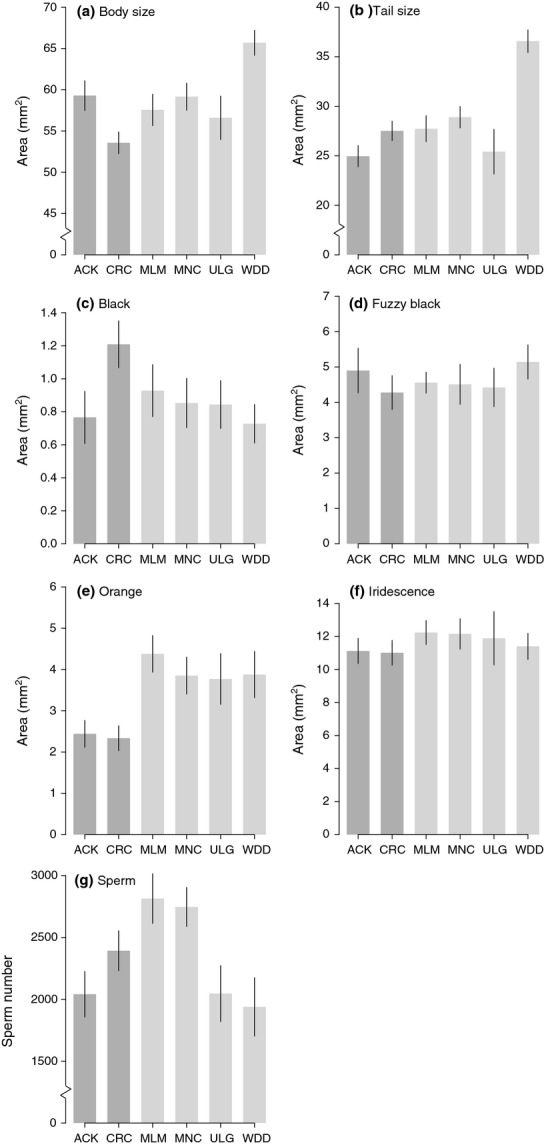
Population means for the male traits measured in the feral guppy populations (acronyms are explained in the main text): (a) body size (body area), (b) tail size (tail area), (c) area of black coloration, (d) area of ‘fuzzy’ black coloration, (e) amount of orange ornamentation, (f) area of iridescent coloration, (g) sperm number. Dark grey shaded populations indicated that predators had been found, whereas the light grey shaded populations have been classified as ‘no predation’. Note that only orange coloration and iridescent coloration were significantly affected by predation and that this remained stable after correction for multiple comparisons for orange coloration only (see Table3 for details).

Multivariate analysis of variance (manova) revealed that male traits differed significantly among populations (Wilk's lambda = 0.386, *F*_5,157_ = 4.633, *P* < 0.001). Univariate analyses showed that these differences were due to large differences in body and tail size, as well as the area of orange coloration and sperm number (body area in mm^2^: *F*_5,157_ = 5.663, *P* < 0.001, tail area in mm^2^: *F*_5,157_ = 11.632, *P* < 0.001, orange area in mm^2^: *F*_5,157_ = 3.783, *P* = 0.003, sperm number: *F*_5,157_ = 3.748, *P* = 0.003, see also Fig.2).

### Sexual selection differs among populations

There were significant differences among populations in linear sexual selection (partial *F*-test: *F*_7,115_ = 4.947, *P* < 0.001). Linear sexual selection gradients, *β*, for the seven traits in each of the six populations are given in Table1 and in Fig. S1.

**Table 1:** Linear selection gradients (*β*) ± SE for each of the seven male traits within each population. Selection gradients in boldface were significant.

	ACK	CRC	MLM	MNC	ULG	WDD
Body	0.322 ± 0.345	−0.888 ± 0.519	**−1.340 **±** **0.564	−0.459 ± 0.803	0.168 ± 0.728	0.051 ± 0.524
Tail	**−1.443 **±** **0.426	0.267 ± 0.478	0.587 ± 0.644	−0.123 ± 0.740	−0.887 ± 0.707	0.467 ± 0.392
Black	0.184 ± 0.245	0.240 ± 0.219	0.125 ± 0.256	0.526 ± 0.397	−0.760 ± 0.530	−0.378 ± 0.379
Fuzzy	0.003 ± 0.227	0.083 ± 0.283	−1.260 ± 0.825	−0.094 ± 0.379	0.522 ± 0.534	0.020 ± 0.323
Orange	0.358 ± 0.381	−0.080 ± 0.413	**0.687 **±** **0.266	0.484 ± 0.482	0.706 ± 0.388	0.106 ± 0.231
Iridescence	0.316 ± 0.364	−0.089 ± 0.363	**1.221 **±** **0.514	0.244 ± 0.483	0.404 ± 0.464	−0.107 ± 0.369
Sperm	−0.139 ± 0.268	−0.088 ± 0.282	0.279 ± 0.244	0.487 ± 0.515	0.701 ± 0.543	0.021 ± 0.263

### Within-population alignment of male trait variation and sexual selection

**Figure 3:**
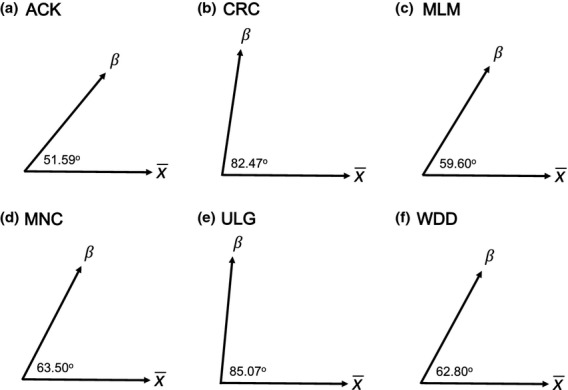
The alignment between phenotypic variation and sexual selection for each of the feral guppy populations, where large angles represent a greater mismatch between trait variation and the direction of sexual selection compared to smaller angles. (a) Alligator Creek, (b) Big Crystal Creek, (c) Mena Creek, (d) Millaa Millaa Falls, (e) Mulgrave River, (f) Wadda Creek.

For each of the six populations, the angle between sexual selection and phenotypic vectors was greater than 50^°^, indicating that there is some form of constraint acting within each population (see Fig.3). By looking at the overlap of the 95% confidence intervals, we can see that the populations formed two groups with respect to the degree of alignment between sexual selection and phenotypic variation. Selection and phenotypic variation were most closely aligned within the Alligator Creek, Millaa Millaa Falls, Mena Creek and Wadda Creek populations (Fig.3), whereas Big Crystal Creek and Mulgrave River had significantly weaker alignment, with intervals overlapping the absolute constraint of 90°. These results suggest that the populations differ in terms of constraints on the evolution of these male traits.

### Among-population covariation in divergence of male traits and sexual selection

The pattern of population divergence in male traits represented by the **D** matrix (Table S1) was explained by two principal components (eigenvectors *d*_max_ and *d*_2_, Table2) that together accounted for 95.2% of the variation among population mean phenotypes. *d*_max_ contrasted body area, tail area and fuzzy black coloration with black coloration and sperm number (Table2). Similarly, most (90.1%) of the between-population variation in sexual selection represented in **B** (Table S2) was also accounted for by variation in two principal components (*b*_max_ and *b*_2_). A comparison of the major subspaces of these two matrices indicated substantial similarity in orientation between them (Σ*λ*_S(B,D)_ = 1.076 of a possible 2, or 53.8% of the maximum).

**Table 2:** Major axes of interpopulation covariance matrices describing observed (D) and predicted (B) divergence due to sexual selection for male traits among six natural populations of guppies.

Trait	D		B	
	*d*_max_	*d*_2_	*b*_max_	*b*_2_
% variation explained	67.67	27.53	56.70	33.44
Body area	−0.456	−0.068	0.493	0.044
Tail area	−0.426	−0.033	−0.427	−0.181
Black	0.424	0.167	−0.396	−0.248
Fuzzy black	−0.457	0.030	0.497	−0.064
Orange	−0.201	−0.642	−0.082	0.627
Iridescence	0.156	−0.673	−0.401	0.376
Sperm no.	0.397	−0.316	0.060	0.604

### The role of predation regime in determining population variation in male traits and sexual selection

The amount of male orange coloration was influenced by the predation regime of the population of origin, whereas males from populations where predators had been observed were less colourful than males from sites where predators were not observed (see Fig.2, Table3). This effect remained significant even after controlling for multiple tests (Table3). Predation regime, however, did not influence the other male traits measured, nor sexual selection acting on any of these (multivariate *F*_1,4_ = 0.176, *P* = 0.924).

**Table 3:** The effects of predation regime on male traits (pooled to population means) and sexual selection gradients (*β*) acting on these traits. Both original and corrected *P*-values are shown. (p_(FDR)_) were calculated using the false discovery rate method to correct for multiple comparisons (Benjamini & Hochberg, 1995).

	Term	*F*_1,4_	*P*	p_(FDR)_
Mean male traits	Body	0.879	0.402	0.697
	Tail	0.848	0.409	0.697
	Black	1.001	0.374	0.697
	Fuzzy	0.049	0.835	0.868
	Orange	56.682	**0.002**	**0.028**
	Iridescence	8.981	**0.040**	0.280
	Sperm	0.223	0.662	0.800

Significant terms are shown in boldface.

### The role of genetic and geographical distance in determining population variation in male traits and sexual selection

Population differences in male traits were associated with genetic distances (Mantel test, *r* = 0.624, *P* = 0.012), but not linear geographical distances (Mantel test, *r* = −0.063, *P* = 0.476). In contrast, differences between populations in sexual selection were not correlated with either genetic distance (Mantel test, *r* = −0.122, *P* = 0.621) or geographical distance (Mantel test, *r* = −0.068, *P* = 0.519).

## Discussion

Guppies from Trinidad, where they occur naturally, provide the most widely cited support for a correspondence between male trait expression and female mating preferences among populations (Endler, 1982; Houde & Endler, 1990; Endler, 1995; but see Houde & Hankes, 1997; Schwartz & Hendry, 2007). Here, we found evidence for a match between male traits and the strength of sexual selection across six introduced Australian guppy populations: more than 50% of male trait variation among populations was due to the variation in sexual selection. This is high compared to the results of a comparable analysis, looking at population divergence in cuticular hydrocarbons of *Drosophila serrata*, which found that only 10% of male trait divergence could be attributed to divergent sexual selection alone (Chenoweth *et al.*, 2010). In *D. serrata*, male trait divergence was highly influenced by the genetic variance–covariance structure, indicating that genetic constraints played a large role. The pattern of multivariate genetic variation in a population strongly influences the trajectory along which each trait evolves (Schluter, 1996; Blows & Hoffmann, 2005), and constrains their evolution. In the guppy populations investigated here, there was considerable variation in the degree of alignment between the direction of sexual selection and that of male trait divergence within populations (ranging between 51° and 85°), suggesting that the potential for evolutionary response in the direction of selection is likely to vary across populations. Genetic constraints are one possible explanation for these results, but further investigation within a quantitative genetic framework would be needed to examine the nature of constraints on male trait adaptation across populations (Blows & Hoffmann, 2005; Blows, 2007).

Most studies relating male trait variation to sexual selection use measures of female preferences. In contrast to this, we estimated sexual selection gradients using paternity data. This provides an overall estimate of sexual selection which incorporates not only female precopulatory choice, but also post-copulatory processes such as female cryptic choice and sperm competition. Such post-copulatory processes have been shown to be important in driving the evolution of male sexual traits in the Alligator Creek population (Evans, 2010). We have shown previously that selection on male attractiveness and female preferences (Brooks & Endler, 2001a; Hall *et al.*, 2004) in the Alligator Creek population is unable to effect appreciable evolutionary change due to multivariate genetic constraint. In our study, Alligator Creek has the best alignment between male traits and sexual selection of all the populations we studied. Thus, the role of genetic architecture in constraining the response of male traits to sexual selection arising from precopulatory choice and post-copulatory processes within populations is likely to be widespread, with the constraints present in other populations investigated here being at least as large as those in Alligator Creek.

Rather than concluding that male trait divergence is due to one process (sexual selection, predator-induced selection or drift), we find evidence that all of them have influenced the observed pattern and that multivariate genetic constraints have also shaped the outcome. The weak but still important fit between sexual selection gradients and male trait divergence may be partially explained by natural selection. Predation has been previously shown to be the most important ecological factor influencing the evolution of male coloration (Millar *et al.*, 2006). In the present study, we also found that divergence in male ornamental traits was associated with differences in the presence/absence of piscivorous predators. However, historical selection regimes in the native source populations may have also played a role in shaping the constraints on the evolution of male traits and female preferences. Although the number of populations we studied was modest, the fact that visual-hunting piscivores appear to reduce both the proportion of males in the population (Head, 2005) and the level of orange coloration provides an interesting parallel with natural and introduced guppy populations within Trinidad. Orange coloration is one of the most consistently implicated cues of mate choice in guppies (Endler & Houde, 1995; Houde, 1997), including Australian populations (Brooks & Endler, 2001a; Blows *et al.*, 2003). Female preferences have been shown to co-evolve more slowly than male ornaments in guppies (Easty *et al.*, 2011) and are known to be highly variable (Zajitschek & Brooks, 2008; Easty *et al.*, 2011), rendering predictions about fine-scaled direction of sexual selection difficult. In addition, female-biased primary sex ratios and reduced courtship and harassment of females by males in the high-predation localities suggest that the relationship between sexual selection and predator-induced selection on male colour patterns may be complex (Head, 2005).

Phenotypic divergence was also correlated with genetic distance measured by neutral markers. It is unclear to what extent genetic distances reflect founder effects, as previous mtDNA analysis from the six populations investigated here point to gene flow or to two female source populations in Guyana and Trinidad (Lindholm *et al.*, 2005). The role of common female founders in explaining trait divergence may be modest, due to the fact that the populations of Alligator Creek and Mena Creek share a single mtDNA type, but did not show similarity in sexual selection. Male-biased gene flow between introduced guppy populations is also likely to have occurred (Lindholm *et al.*, 2005), but the effects of gene flow and admixture of founder populations cannot be fully disentangled.

While some Trinidad populations have been separated for 200 000 years (Fajen & Breden, 1992), the North Queensland populations have only been introduced in the last century. Despite this short time frame, and bottlenecks which have reduced genetic diversity (Lindholm *et al.*, 2005), sexual selection differs substantially between the populations in ways that have shaped male sexual trait variation. Theoretic models of mate choice evolution show, however, that in the vast majority of circumstances, direct selection on choice and signal will swamp the indirect co-evolutionary processes that cause an association between trait and preference (Kokko *et al.*, 2006). Direct selection on male ornamentation in a new environment is likely to initially involve direct adaptation to the signalling environment including signal propagation considerations and the presence of predators (Endler, 1987). Likewise, direct selection on choice might also be shaped more by factors such as predators and food in a new environment than by the more subtle effects of signaller–receiver co-evolution. Further, in guppies, heritabilities of male traits are much higher than heritabilities of female choice (Houde, 1992; Brooks & Endler, 2001a,b; Hall *et al.*, 2004; Hughes *et al.*, 2005), suggesting greater potential for male traits to respond rapidly during introduction to new environments.

## Conclusions

Here, we show that populations of recently introduced guppy populations differ significantly in both male sexual traits and sexual selection on these traits. Furthermore, we show the existence of substantial among-population covariation between sexual selection and male traits. We thus demonstrate that differences in sexual selection between populations are an important driver of population variation in male traits, despite the effects of other factors (e.g. ecological selection, evolutionary history) that are expected to constrain evolutionary responses. Our results may have important implications for understanding how sexual selection contributes to population divergence and speciation. In addition, the rapid divergence in male traits under different ecological conditions highlights how introduced species are likely to adapt to new environments. Further studies determining the generality of our results in other systems, as well as studies that incorporate quantitative genetic breeding designs, will be important next steps for research on how organisms adapt to new environments and how sexual selection contributes to trait divergence between the populations.

## Supporting Information

Figure S1 Linear selection gradients for each male trait in the feral guppy populations in North Queensland: Alligator Creek (‘Ack’), Big Crystal Creek (‘Crc’), Mena Creek (‘Mnc’), Millaa Millaa Falls (‘Mlm’), Mulgrave River (‘Ulg’), Wadda Creek (‘Wdd’).

Table S1 Interpopulation variance–covariance matrix among-population means (**D**) for the seven male traits for the six natural populations of *Poecilia reticulata*. **Table S2** Interpopulation variance–covariance matrix of directional sexual selection gradients (**B**) acting on the seven male traits for the six natural populations of *Poecilia reticulata*.
