# Supplementary figures and images for: Causes of male sexual trait divergence in introduced populations of guppies
Source: J Evol Biol. 2014 Jan 23;27(2):437–48. doi: 10.1111/jeb.12313 (PMC4237193; doi:10.1111/jeb.12313)

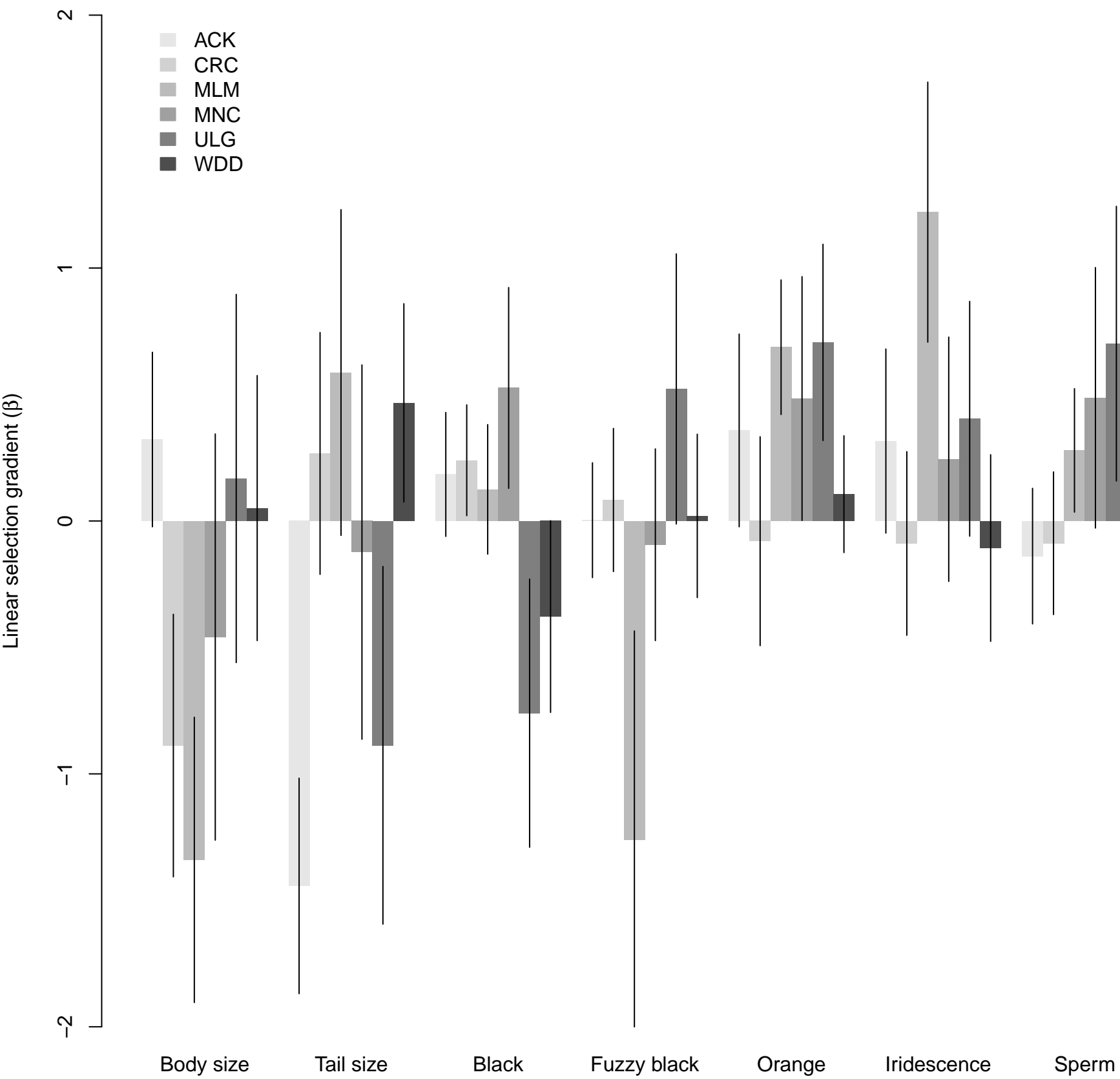

Supplement: Figure S1 — Linear selection gradients for each male trait in the feral guppy populations in North Queensland: Alligator Creek (‘Ack’), Big Crystal Creek (‘Crc’), Mena Creek (‘Mnc’), Millaa Millaa Falls (‘Mlm’), Mulgrave River (‘Ulg’), Wadda Creek (‘Wdd’). [file jeb0027-0437-SD1.pdf]
