# Supplementary material for: Causes of male sexual trait divergence in introduced populations of guppies
Source: J Evol Biol. 2014 Jan 23;27(2):437–48. doi: 10.1111/jeb.12313 (PMC4237193; doi:10.1111/jeb.12313)
Supplement: Table S1 — Interpopulation variance–covariance matrix among-population means (D) for the seven male traits for the six natural populations of Poecilia reticulata. Table S2 Interpopulation variance–covariance matrix of directional sexual selection gradients (B) acting on the seven male traits for the six natural populations of Poecilia reticulata. [file jeb0027-0437-SD2.docx]

Table S1. Inter-population variance-covariance matrix among-population means (**D**) for the seven male traits for the six natural populations of *Poecilia reticulata*

|  | Body | Tail | Black | Fuzzy | Orange | Iridescence | Sperm |
| --- | --- | --- | --- | --- | --- | --- | --- |
| Body | 0.168 |  |  |  |  |  |  |
| Tail | 0.178 | 0.304 |  |  |  |  |  |
| Black | -0.074 | -0.037 | 0.050 |  |  |  |  |
| Fuzzy | 0.046 | 0.041 | -0.021 | 0.015 |  |  |  |
| Orange | 0.055 | 0.071 | -0.031 | 0.005 | 0.121 |  |  |
| Iridescence | 0.001 | -0.003 | -0.006 | -0.003 | 0.035 | 0.013 |  |
| Sperm | -0.057 | -0.037 | 0.033 | -0.023 | 0.045 | 0.026 | 0.128 |

Table S2. Inter-population variance-covariance matrix of directional sexual selection gradients (**B**) acting on the seven male traits for the six natural populations of *Poecilia reticulata*

|  | Body | Tail | Black | Fuzzy | Orange | Iridescence | Sperm |
| --- | --- | --- | --- | --- | --- | --- | --- |
| Body | 0.440 |  |  |  |  |  |  |
| Tail | -0.395 | 0.661 |  |  |  |  |  |
| Black | -0.147 | 0.059 | 0.221 |  |  |  |  |
| Fuzzy | 0.302 | -0.260 | -0.119 | 0.357 |  |  |  |
| Orange | -0.006 | -0.069 | -0.033 | -0.054 | 0.100 |  |  |
| Iridescence | -0.162 | 0.035 | 0.014 | -0.224 | 0.120 | 0.235 |  |
| Sperm | 0.001 | -0.018 | -0.057 | 0.023 | 0.081 | 0.057 | 0.114 |

Figure S1: Linear selection gradients for each male trait in the feral guppy populations in North Queensland: Alligator Creek (“Ack), Big Crystal Creek (“Crc”), Mena Creek (“Mnc”), Millaa Millaa Falls (“Mlm”), Mulgrave River (“Ulg”), Wadda Creek (“Wdd”).
